# Relationship between Pain, Somatisation, and Emotional Awareness in Primary School Children

**DOI:** 10.1155/2018/4316234

**Published:** 2018-11-12

**Authors:** M. Rossi, G. Bruno, M. Chiusalupi, A. Ciaramella

**Affiliations:** ^1^Aplysia Onlus, GIFT Institute of Integrative Medicine, Pisa, Italy; ^2^Department of Surgical, Pathology and Clinical Area, Pisa, Italy

## Abstract

Poor emotional awareness (EA) seems to play an important role in the aetiology of functional somatic complaints featuring pain as a form of somatisation. The aim of this study was to shed more light on this relationship by investigating the links between pain, somatisation, and emotional awareness in a nonclinical population of 445 children aged 6–10. Assessing pain through the Children's Somatisation Inventory (CSI), a very high percentage of the entire sample complained of experiencing pain at least one site (84.07%) over the preceding 2 weeks. Although no difference in the prevalence of pain (whole) was found when the sample was subdivided by Levels of Emotional Awareness Scale-Children (LEAS-C), a relationship between low level of LEAS-Self and prevalence of headache (H) was detected (*χ*2=7.69, p=0.02). LEAS (Self) was correlated with the intensity of back pain (BP) (r=-0.12; p< 0.05), H (r=-0.12; p< 0.05) but not with abdominal pain (AP). Pain worsened QoL, and the greatest negative correlation with total KidScreen-10 was found for abdominal pain (r=-0.14; p< 0.01). Our results suggest that low EA is a predictor of somatisation, BP severity, H, and severity of pain in general, but not AP.

## 1. Introduction

The DSM-5 criteria classify somatic symptoms disorder with predominant pain (SSP) as belonging to the category: “Somatic Symptom and Other Related Disorders” [1]. Before the advent of the DSM-5, somatisation was often described as medically unexplained pain (MUP), i.e., pain whose cause could not be medically determined and for this reason was perceived as a somatisation disorder. However, episodic MUP is not sufficient for a diagnosis of somatic pain disorder (SSP), and it can not be considered a symptom of somatisation. Indeed, SSP needs to be distressing or results in a significant disruption of functioning due to excessive and disproportionate symptom-related thoughts, feelings, and/or behaviours.

Pain is one of the most frequent forms of somatisation, and somatic pain is thought to be the expression of a distress that cannot be conveyed verbally. However, somatic pain is not the same thing as SSP, despite sharing a common pathogenetic mechanism: the expression of distress through physical pain. Indeed, somatic pain tends to be episodic and not persistent (as in some cases of SSP), and SSP may only be diagnosed when it persists for 6 months or more.

2%–10% of the general paediatric population complains of aches and distressing pain (stomach, abdomen, headache, and joints). However, these are likely to be medically unexplained and are often transient and do not affect overall functioning [2]. Recurrent distressing somatic pain has been investigated in an Italian paediatric emergency department; 8.6% of children complaining of pain were assessed as having SSP, approximately 25% of school-aged children experience chronic or recurrent pain (e.g., headache, abdominal pain, and sore muscles) and 10% report chronic fatigue [3]. One of most common pain complaints in children is abdominal pain (AP), and an annual 2–4% of primary paediatric care appointments are for AP [4], while 10% to 19% of children complain of this pain at school [5]. Low back (LB) pain is also frequent in childhood, with an estimated prevalence ranging from 13 to 51%, while recurrent LB pain is reported in from 7 to 27% of school-age children [6–8].

However, very few studies have been conducted into the links between pain and somatisation in children and adolescents, and those that have been published do not shed much light on many factors regarding epidemiology and disability in this age group. Nonetheless, several authors have claimed that recurrent pain during adolescent years may be a precursor of chronic LB pain in adults [8, 9]. In children and adolescents, LB pain and AP are often medically unexplained, and together with stomach ache and joint pains account for as many as 50% of new medical outpatient appointments in this population [10]. Although these epidemiological studies do reveal the great impact of pain in the paediatric population, there is persistent confusion as regards differential diagnosis between pain as an expression of somatisation (with distress), MUP (not necessarily accompanied by distress), recurrent pain, and chronic pain (persistent for at least 3 months) [11].

When pain cannot be explained by medical issues, is not intentionally produced or simulated, and is associated with certain psychological factors, it can be considered, to all intents and purposes, somatisation [10]. The most frequent psychological problems associated with somatisation are feeling low, irritability or bad temper, difficulty sleeping, and nervousness [12], but studies into childhood somatisation have generally relied on parental reports [13], even though pain is a subjective experience that is more effectively reported by the patient themself [14].

To this end, a promising international self-report instrument is the Children's Somatisation Inventory (CSI), which was developed specifically to assess the occurrence of somatisation symptoms in children and adolescents. The first CSI version featured 36 items and included several symptoms based on the DSM III-R Somatisation Disorder [15]. Although this version incorporates several symptoms that are generally absent in children (e.g., sexual and heart-related), demands from the scientific community led to the production of a shorter tool (24 items), which includes several pain symptoms and has proven to be fairly internally consistent and reliable, even for children aged around 7 years [16–18].

Using this type of assessment, some studies have shown a significant relationship between somatic symptoms (SS) and alexithymia, a psychological factor abundantly investigated in chronic pain [19] in both adults [20] children [21]. Poor emotional awareness and the alexithymia personality trait seem to play an important role in the aetiology of functional somatic complaints featuring pain as a form of somatisation. Adults with alexithymia have difficulty in identifying and describing their feeling and emotions, mirroring the difficulty children and adolescents often have in expressing their feelings and emotions through language; in such cases psychological distress may be expressed through somatic symptoms such as pain.

More recently, the concept of emotional awareness (EA) has benefitted from special attention in the scientific community. Poor EA includes the essential components of alexithymia, as well as difficulty in recognising, identifying, and correctly labelling emotions in others [22], and research in adults and children has shown that alexithymia and poor EA are significantly associated with somatic complaints [23–26]. The relationship between pain, somatic complaints, alexithymia, and emotional awareness has been confirmed by functional neuroimaging studies, which have revealed the activation of connectivity of the anterior cingulate cortex (ACC) and anterior insula (AI), Prefrontal Cortex (PFC) as the cornerstone of this relationship [27, 28].

Based on these findings, it may be that a reduction in emotional awareness in the age range 6–10 years, just before preadolescent modification of the brain begins, may increase the likelihood of the onset of alexithymia, predisposing the adult to somatisation and pain. Hence, the principal aim of this study was to shed more light on this relationship by investigating the links between pain, somatisation, and emotional awareness in a nonclinical population of primary school-age children. We also set out to determine whether this relationship changes with gender and/or age and if somatisation expressed as pain affects quality of life in such children.

## 2. Methods

### 2.1. Sample and Procedure

**Figure 1:**
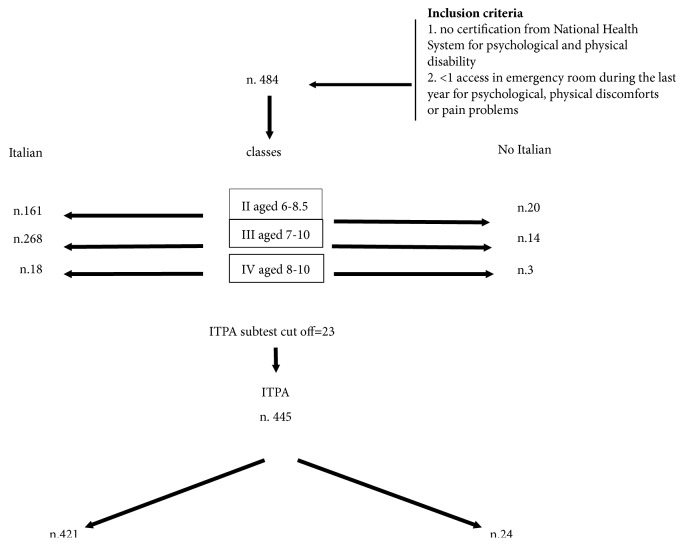
Flowchart of sample selection.

This is a retrospective study performed as part of a project entitled “Experiencing Emotion through the Body” (EETB), run by a not-for-profit association (Aplysia Onlus). EETB is a psychoeducation project on emotional awareness for the primary prevention of somatoform disorders. EETB baseline data collected in 9 primary schools in Tuscany, Italy, over a period of 6 months was analysed for this study. To meet legal and ethical requirements, the not-for-profit association drew up and signed a formal contract with each school clearly describing the aims and methodology of the EETB project. The contract also contained information explaining that the project was part of the educational aims of the university (training for traineeships), and that its data may be disseminated for scientific purposes. The headteacher and teacher of each class were responsible for informing the parents about their children's participation in the project. The children of any parents who refused consent were excluded from the study. All exclusion and inclusion criteria for the study were established at the first meeting with the school headteacher. All children with psychological and/or somatic disabilities certified by the National Health System were excluded, as were children with more than 1 admission to the emergency room for psychological issues and/or physical discomfort or pain. All children of nationalities other than Italian were considered for inclusion if they had been domiciled in Tuscany for at least 6 months. Selection of both Italian and non-Italian participants was conditional upon their obtaining a score >23 on the Illinois Test of Psycholinguistic Abilities (ITPA) “verbal expression” subset. This cut-off represents the average score calculated for the Italian children in the n. 484 children of original sample minus the standard deviation. No information was collected for children who did not meet the selection criteria (Figure 1) or failed to complete one or more of the questionnaires.

The research was conducted in accordance with Declaration of Helsinki ethical principles for medical research involving human subjects, and the anonymity of participants was protected. Being a retrospective study, no Ethics Committee approval was necessary. In accordance with the educational aims of the EETB, parents of participating children were shown a PowerPoint presentation of the collective outcomes (no individuals were identified).

### 2.2. Instruments

#### 2.2.1. Levels of Emotional Awareness Scale-Children (LEAS-C)

Emotional awareness (EA) has been defined as the ability to identify, label, and describe individual emotions [29]. It is a fundamental skill, essential for the proper psychological, emotional, and social development of an individual. Lane and Schwartz [30] proposed that an individual's ability to recognise and describe emotion in oneself and others is a cognitive skill that undergoes a developmental process similar to that which Piaget described for cognition in general. Accordingly, their cognitive-developmental model posits five “levels” of emotional awareness, which share the structural characteristics of Piaget's stages of cognitive development. In ascending order, these five levels of emotional awareness are physical sensations, action tendencies, single emotions, blends of emotion, and blends of blends of emotional experience. Based on this theory, Lane and colleagues went on to develop the Levels of Emotional Awareness Scale (LEAS) [31], which measures the level of awareness regarding an adult's own and others' emotions. Subsequently [32], they also constructed a version suitable for the evaluation of emotional awareness in developing subjects. This format is generally recommended for children of 8 years of age or younger, but in several studies it has been administered to children older than 8; in fact, as reported in the supplemental LEAS scoring manual, although LEAS-C was designed for self-reporting it can be administered in an interview format to groups, and orally to children younger than 8 [33, 34]. We included children aged 6–10, provided that they exceeded the ITPA subset score of 23. Indeed, the ITPA has been validated in 6–year-olds [35], and this criterion excluded children with below average language comprehension and expression.

The LEAS-C comprises 12 scenarios based on everyday social situations (mainly school- and home-related). Each scenario involves two people, the respondent, and another person, and after each scenario is described, respondents are asked two questions: “How would you feel?” in this situation and “How would the other person feel?” Children are required to generate their own answers to the questions. Scoring procedures for the LEAS-C are the same as those used for the adult-based LEAS, and the complexity of emotional awareness is assessed on a 5-point scale ranging from 0 to 4. Three scores are allocated for each scenario: Self Awareness, Other Awareness and Total Awareness. Total scores depend on the degree of differentiation between the emotional states of the “self” and “other”. The total score equals the highest score obtained for “self” or “other”, when no differentiation is made, while it equals 5 when differentiation is clearly apparent. A glossary of words accompanies the scoring manual to aid in the scoring of emotion words.

In the present study we used the LEAS-C Italian version developed by Marchetti and coworkers [36], who reported that the cognitive abilities of EA increase after age 8, and are dependent on gender and language skills. In order to verify the internal consistency of the LEAS-C, the Italian researchers calculated the Cronbach's alpha for each scale. The results, “Self” scale *α* = .704; “Other” scale *α* = .669; “Total” scale *α* = .713 (N = 125), indicate that the scale possesses good reliability and internal consistency.

#### 2.2.2. The Children's Somatisation Inventory (CSI)

The CSI, specifically the short version (CSI-24) [17, 37], which was translated into Italian by Cerutti and coworkers [21] using the translation/back-translation method, was used to assess each child's perception of somatic symptoms (SSD). This instrument is one of the most commonly used to assess somatisation among children and adolescents [38]. The CSI-24 score was computed by summing items, as reported by Walker and colleagues [17, 37] in the accompanying instructions in Appendix I. Items are scored 0–4 for all 24 items (0=not at all, 1=a little, 2=somewhat, 3=a lot and 4=a whole lot), and item sum scores range from 0 to 96. The CSI has demonstrated adequate reliability and validity, and in healthy paediatric samples the internal consistency (i.e., Cronbach's alpha) of the CSI-24 was .87 [17, 37]. Although in the Italian version the Cronbach's coefficient of .84 indicates good internal consistency, no explicit validation of the Italian instrument as administered to paediatric patients with chronic abdominal pain is mentioned in the article [21].

In the present study, pain symptoms were extrapolated from the CSI-24 (items 5, 3, 24, 15, 1, 6) and scored according to Walker and colleagues' instructions [17, 37], and via dichotomous scoring in which 0 indicates the absence of a symptom and 1 indicates its presence (i.e., when the Walker score is 1–4). The assessment refers to the previous 2 weeks.

#### 2.2.3. The KidScreen-10

The KidScreen project, promoted by the European Union, aimed to produce self-disclosure quality of life (QoL) questionnaires for healthy and chronically ill children and adolescents, giving due weight to cultural issues [39, 40]. This health-related quality of life Questionnaire (HRQoL) only includes items representative of a global unidimensional latent trait. Several versions of KidScreen (self-report and proxy versions with 52, 27, and 10 items) were simultaneously developed in 13 different European countries in order to ensure cross-cultural applicability, using methods based on classical test theory. In this study we used the Italian version of KidScreen-10—a self-report scale containing 10 items [41] which has shown reliable internal consistency (Cronbach's alpha = .82) and good test–retest reliability/stability (r = .73; ICC=.72) [40]; each item is answered on a 5-point response scale exploring the level of the child's/adolescent's physical activity, energy and fitness, depressive moods and emotions, and stressful feelings. Other items explore opportunities to structure and enjoy their social and leisure time, and participation in social activities, interaction between the child/adolescent and their parent or career, and the child's/adolescent's feelings towards their parents/careers, as well as the nature of the child's/adolescent's relationships with other children/adolescents, and perception of their cognitive capacity and satisfaction with school performance. In this study, we used the raw scores represented by the total sum of the scores of the 10 items. This assessment makes reference to the preceding week.

#### 2.2.4. The Illinois Test of Psycholinguistic Abilities (ITPA)

Kirk, McCarthy, and Kirk [42, 43] developed the ITPA, based on Osgood's psycholinguistic model [43], to measure the intraindividual visual-motor and auditory-vocal strengths and weaknesses of children. The ITPA is an effective measure of children's spoken and written language and consists of 12 subtests, each measuring some aspect of language, including oral language, writing, reading, and spelling. The ITPA provides different composite scores for clinical and diagnostic use, and in the present study we used the “general language composite” score, which combines the results of all 12 subtests (10 fundamental and 2 optional) [35]. According to the authors, this score is the best single estimate of linguistic ability, because it reflects the widest range of spoken and written language. The ITPA presents good psychometric features: reliable internal consistency, (0.87), stability (0.87), and validity [42]. Each subset also has good internal consistency, and the flexibility of the instrument allows its use in various ways and in different conditions. In particular, the ITPA is generally used in 6-year-olds, but may also be used in children aged 8 [35]. In our study we used only the “verbal expression” subset with normative scoring of the Italian version of the ITPA [44]. This subset consists of showing 4 objects: ball, cube, envelope and button; children are asked to freely describe each object using 10 categories, of which 5 are considered essential (label, colour, shape, material, function). If the free description does not include the essential categories, the interviewer asks specific questions to elicit them. If child's description of the object still lacks an essential category, the score will be 0.

### 2.3. Statistical Analysis

All data were analysed using IBM SPSS Statistics 21. First, means and standard deviations (sD) of the demographic data and total scores of the 4 instruments used and of the CSI items assessing pain were calculated. Then, after the application of the Kolmogorov-Smirnov test—which gives details about the Gaussian distribution of the data—Pearson correlation analysis was performed; correlation coefficients > 0.10 were considered statistically significant (for Cohen's standard this is a low effect size). After determining the correlation among variables, we next investigated if emotional awareness can be considered a predictor of somatisation and pain when considered as somatoform symptoms. To this end, a stepwise multiple regression analysis was performed using quality of life (total of KidScreen 10 scoring), somatisation (CSI total scoring) and intensity of pain at each site investigated as dependent variables, and LEAS-Self, LEAS-Other, LEAS-Total, and Language (ITPA verbal expression subtest) scores as independent variables. Statistical significance was set at p<0.05.

## 3. Results

The inclusion criteria and demographic features of the sample are reported in the flowchart in Figure 1. Of the 484 children initially enrolled (Supplementary Table (available here)), only 445 exceeded the ITPA verbal expression subtest score cut-off of 23; this was calculated by subtracting the standard deviation (5.89) from the mean ITPA subtest score of the original population, which was 29.20 (range 8–52).  Table 1 provides a description of the variables pertaining to the 445 primary schoolchildren (242 males and 203 females) aged 6–10 definitively enrolled in this study.

**Figure 2:**
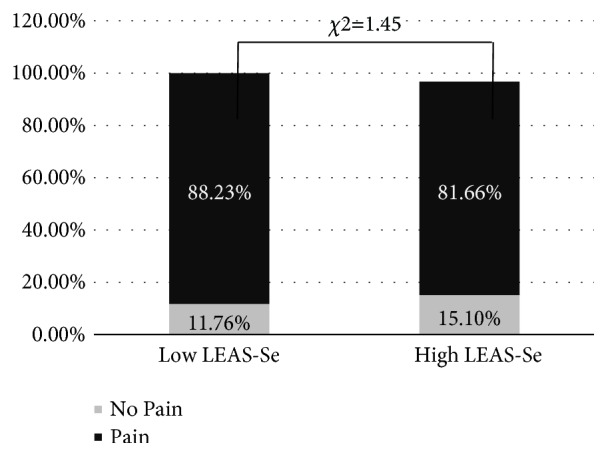
Difference in prevalence (percentage) of pain (total) in the sample split into two groups by LEAS score: two groups: less than the mean (Low LEAS-Se) and equal to or greater than the mean (High LEAS-Se).

**Figure 3:**
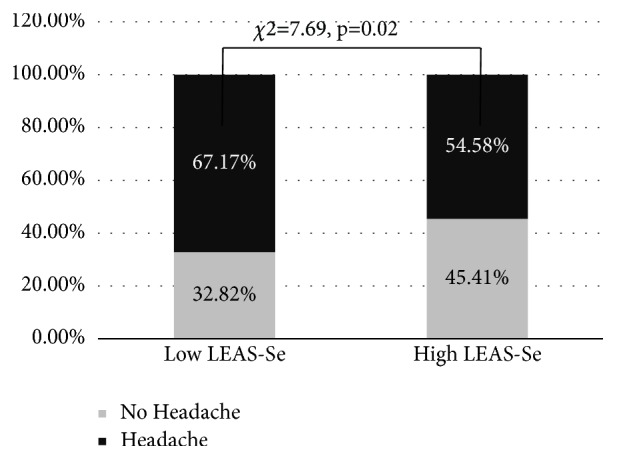
Difference in prevalence (percentage) of headache in the sample split into two groups by LEAS score: two groups: less than the mean (Low LEAS-Se) and equal to or greater than the mean (High LEAS-Se).

A very high percentage of this sample complained of experiencing pain at least one site (84.07%) over the preceding 2 weeks (Table 1). Although the pain was not described as very intense (the highest CSI score was 3, not 4), children often complained of pain at more than one site (mean=2.75, sD=8.63). As far as the type of pain was concerned, we found the highest prevalence for headache (H) (58.20%), followed by limb pain (LP) (56.17%) and abdominal/stomach pain (AP) (54.83%). No difference in the prevalence of pain (as a whole) was found when the sample was subdivided by mean LEAS-Self score. Unexpectedly, the group of children with lower mean LEAS-Self scores (Low LEAS-Self) did not show any greater prevalence of pain with respect to the group with LEAS-Self scores equal to or greater (High LEAS-Self) than the mean (*χ*^2^=1.45) (Figure 2). Nonetheless, the LEAS-Self mean scores were associated with the prevalence of headache symptomatology, which was greater in the Low LEAS-Self than the High LEAS-Self group (*χ*2=7.69, p=0.02) (Figure 3).

As can be seen in Table 2, total CSI scores were strongly correlated with the intensity of pain at all sites investigated, but there was no statistically significant difference (t=1.57) in total CSI score between Low LEAS-Self (mean CSI 15.72, sD=8.96) and High LEAS-Self (14.32, sD=8.21). However, as reported in the literature, Language (ITPA “verbal expression” score) was correlated with LEAS-Self and especially LEAS-Total, and we also found that Language was correlated with widespread pain, sore muscles and H, but not with CSI (Table 2). The correlation between ITPA scores, LEAS-Self, and LEAS-Total underlines the close link between the EA and language. For this reason we included the ITPA subtest score as a dependent variable in the multiple regression analysis model, but also assessed its value with LEAS as a predictor dimensions (independent variable).

### 3.1. Effect of Age and Gender on the Relationship between Emotional Awareness, Pain, and Somatisation

**Figure 4:**
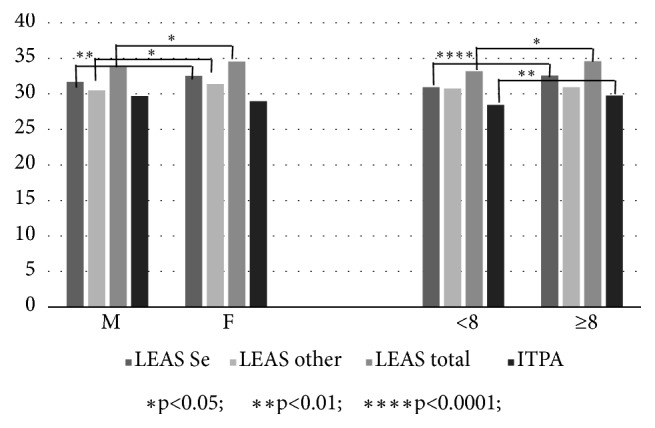
Differences in emotional awareness and language (ITPA verbal expression subset) scores between children split in gender and age groups.

EA increases with age. LEAS-Self and LEAS-Total scores were positively correlated with age (Table 2). As reported by Marchetti and coworkers [36], emotional awareness (EA) increases significantly at the age of eight, when almost all children seem to have reached emotional awareness maturity (as shown by the results of their LEAS testing). The same studies showed higher EA scores in female children. Based on this premise, we divided our sample into two groups, first based on gender and then on age, i.e., those aged less than 8 years old (<8), and those with an age equal to or greater than 8 years (≥8). Our results revealed that females score higher than males on LEAS-Self (t=1.98; p=0.047), LEAS-Other (t=1.97; p=0.049), and LEAS-Total (t=1.96; p=0.050), and that scores for LEAS-Self (t=3.59; p<0.0001) and LEAS-Total (t=3.11; p=0.002) were significantly higher in children aged ≥8 (Figure 4). Unlike similar studies in adults, we found no striking differences between genders in terms of pain perception in children. Likewise, no differences in the severity of pain as a somatisation symptom or total somatisation scores (global CSI) were found when the sample was split on the basis of gender (M versus F) or age (<8 versus ≥8 years). However, the ≥8-year age group showed increased ITPA “verbal expression” scoring with respect to younger children (t=2.04; p=0.041) (Figure 4). No difference in the prevalence of pain as a somatisation symptom was found between the two groups allocated on the basis of age (*χ*2=0.124).

### 3.2. Emotional Awareness as a Predictor of Pain and Somatisation

Using stepwise multiple regression analysis, we found that LEAS-Self is an important predictor of somatisation and pain, when this is considered as a symptom of somatisation. The first model included LEAS-Self as predictors, and global CSI, severity of pain in general, and intensity of back pain and headache in particular as dependent variables (or outcome variables) (Table 3). One stepwise analysis model identified LEAS-Self as the only negative predictor of severity of pain and somatisation symptoms. As shown in Table 3, EA was the only predictor of BP intensity; LEAS-Self was a stronger predictor when associated with LEAS-Total in a second model (r=.247). A similar relationship was detected when the severity of total pain was considered; in other words, this model linked LEAS-Self with LEAS-Total as a predictor for total severity of pain (r=.204).

Interestingly, our data also showed that language may be associated with pain as a symptom of somatisation. In particular, a model that included “verbal expression” ITPA subset scores indicated a link between language and the severity of muscle pain and widespread pain (Table 3); better verbal expression skills was found to be an important predictor of widespread pain, both alone and together with Low LEAS-Self scores, as indicated by the fourth stepwise regression analysis we performed. Similarly, a close relationship between language and emotional awareness was suggested by 2 models for predicting the severity of headache, and 1 model pertaining to muscle pain. A link between LEAS-Total and ITPA “verbal expression” was also found in one stepwise multiple regression analysis model, which showed that LEAS-Total is a positive predictor of better verbal expression (Table 3).

### 3.3. Impact of Pain as Somatisation on Quality of Life

The KidScreen-10 measures the general HRQoL, including items to investigate physical activity, energy and fitness, depressive moods and emotions, stressful feelings, and family and social relationships. Our data did not reveal an association between total KidScreen-10 scores (tKS10) and EA (Table 2). Moreover, EA and language did not predict poor HRQoL according to stepwise multiple regression analysis (Table 3). Unsurprisingly, widespread pain, and the intensity of abdominal pain and headache were negatively correlated with HRQoL, but these relationships were independent of EA (Table 2). The greatest negative correlation with tKS10 was found for abdominal pain.

## 4. Discussion

Several studies on adult and child samples have reported a link between pain and alexithymia [19–21], but very few have investigated the relationship between somatisation, pain and emotional awareness. A study by Zunhammer and coworkers [45] demonstrated a reduction in emotional awareness associated with an increased alexithymia score in adult subjects with “Pain Disorder associated with Psychological Factors” (according to DSM IV criteria) with respect to a healthy control group. However, in one study of an adult population with somatoform disorder, assessed using ICD 10 criteria, Subic-Wrana et al. [23], showed a significantly lower level of EA—with a very high effect size (Cohen's d 0.95)—in subjects with somatoform disorder with respect to a control group.

Although several papers have investigated pain in children, and another alexithymia in children with somatoform disorder and autism [46, 47] not much research into the relationship between emotional awareness (which is an essential component of alexithymia) and pain (considered one of major symptoms of somatisation) in children have been published. However, investigation of emotional awareness in children approaching preadolescence (6–10 years) could clarify the factors influencing the onset of alexithymia, and how these factors can affect the alexithymia-pain relationship. Indeed, the important changes in brain structure that occur in preadolescence could stabilise relationships (e.g., between low emotional awareness and the presence of somatic pain), creating a pathological predisposition to stable dysfunctional personality traits (e.g., alexithymia) and facilitating the onset of somatisation symptoms (including pain). Hence, we set out to investigate the relationship between pain, somatisation and EA in primary school children aged 6–10 years. The hypothesis was that our nonclinical sample (no certified psychological or physical disability and ≤1 emergency room admission for psychological, physical or pain issues during the last year) could provide us with important information about children's predisposition to alexithymia, EA being an essential component of alexithymia, [22] minimising the risk of bias due to the presence of physical and mental disorders.

Although we found no relationship between EA and the prevalence of pain symptomatology (Figure 1), we did detect a negative correlation between EA levels and a greater degree of somatisation (CSI total scoring), as well as the severity of pain in general, and the severity of LB and headache in particular (Table 2). As already mentioned, our results confirm findings by previous research conducted in the adult clinical population. Our finding that low emotional awareness affects the intensity of pain and the tendency to somatise indicates that emotional awareness may be involved in the ability to estimate and discriminate pain. This would imply that EA is the direct expression of the activity of the higher functions that involve the ability to integrate pain perception. Indeed, some studies have reported a role for empathy in the construct of EA [23, 28]. Empathy is a complex emotional and social phenomenon characterised by the ability to understand another's emotional state; it is made up of two main components: affective sharing and mentalising processes, mentalising being the ability to make inferences about the mental state of others [23, 30, 31]. The activity of the anterior cingulate cortex (ACC) and the anterior insula cortex (AIC) increases when subjects experience emotions, and AIC activation in particular is associated, other than subjective sensations from the body, with empathetic feelings [27]. Furthermore, the insula is the site of integration between sensory input from the spinal cord (posterior insula) and the higher mentalisation functions of connections with the prefrontal cortex (anterior insula) [27]. A reduced integration of sensory information (including an increase in pain perception) could be determined by a dysfunction of prefrontal cortex-insula connectivity, consistent with a modification of the posterior to anterior gradient of the insula towards greater behavioural complexity (like empathy) in the frontal cortex. This phenomenon could be expressed through the reduction of EA.

In fact, we also found an association between low-level EA and the prevalence of headache (Figure 2). This indicates that both the onset and severity of headache, as a somatisation symptom, is strongly linked to the degree of EA. Indeed, a relationship between emotion, EA and idiopathic headache has been proposed by Bussone and Grazzi [48], who posited the mechanism of pain as a part of an emotional response induced by alterations in the homeostasis of the interoceptive system that integrates nociceptive information with the emotional network (mediating emotional awareness). From this perspective, EA would be the substrate that represents a vehicle for integrating interoceptive information with headache. Our data demonstrate that the relationship between EA and headache is already evident at an early age, and is also present in conditions in which the full-blown disorder has not yet become established.

In addition to the large influence of LEAS on severity of pain, another important finding from our data analysis is that back pain is strongly influenced by low levels of EA. This was confirmed by Pearson correlation analysis (Table 2). Jones et al. [6], in their work, investigated schoolchildren (older than ours) via point prevalence analysis, finding that 15.5% had recently experienced low back pain, but that this did not lead to disabling consequences. Our data confirm these findings (Table 2), suggesting that low EA is a psychological condition associated with greater low back pain, considered as a symptom of somatisation, but does not worsen quality of life. In contrast, the severity of abdominal/stomach pain—common in our children—worsened QoL (Table 2), even though it does not appear that a deficit in EA is associated with this symptom, and therefore does not appear to be the psychological factor associated with AP somatisation.

Widespread pain, AP, and headache seem to be the pain symptoms which cause the greatest deterioration in quality of life. In our results, however, we found no impact of total somatisation (global CSI score) on QoL, partially confirming the findings by Garralda [2] that overall functioning is preserved in children with somatic symptoms. Contrasting with that study, however, our results indicate that aches and pain in the stomach, abdomen, and head, associated with distress, do affect QoL in children (Table 2).

As shown in Table 3, EA is an important predictor of somatisation and the severity of pain in general and of BP and headache in particular. In addition to predicting somatisation in general, our data demonstrate that lower levels of self-EA predispose children to perceive back pain and headache as more intense. Our data also indicate that language alone and in association with EA too are important predictors of the severity of low back pain as well as of headache and widespread pain. Language, on the other hand, does not seem to predict somatisation in general (global CSI).

From these results it emerges that, in addition to an affective component, a cognitive component also seems to be involved in the severity of pain, especially lumbar pain and headache. The relevance of language in the perception of pain was underscored in 1971 by Melzack and Torgenson [49], whose main objective was to describe the quality of pain. In 1986, Jerrett and Evans [50], among other studies, investigating the appropriateness of pain description by children, showed that pain terminology appears at a very early age [51]. We, on the other hand, reveal a very close link between language and EA, and that LEAS-Total appears to be a strong predictor of increased verbal expression scores (ITPA) (Table 3). The LEAS-Total score is, in fact, more than the mere sum of the LEAS-Self and LEAS-Other dimensions; it increases greatly when children are able to describe in a very detailed way what they feel about themselves and others in the LEAS scenarios. There may be some neurobiological explanation for our findings. After Damasio and Damasio [52], several other studies have investigated the brain area involved in language, and some have demonstrated the involvement of the prefrontal cortex (dorsolateral prefrontal cortex or DLPFC) in sentence comprehension [53]. The DLPFC also appears to be involved in the encoding of acute and chronic pain, as a part of a neuromatrix [54], but prefrontal areas also participate in the presentation of mental states of the self and others as part of emotional awareness according to the Lane model [55]. Hence, a low level of emotional awareness in childhood may modify the activity of the prefrontal area, predisposing an individual to increased pain perception and somatisation. Indeed, this age range precedes preadolescence, which represents a critical period in terms of the supraspinal control of pain [56].

Although this study brings some new findings, there are several limitations to note. First and foremost, we did not obtain informed consent directly from the parents, relying instead on a formal contract signed by the school. Nevertheless, the school headteacher informed all parents about the project, and no child who opted out was included. The parents of child participants were also informed of the collective results of the study in a dedicated meeting.

Another limitation of our methodology is that some of the questionnaires we used were designed for use in older children. We attempted to ensure that the younger children's language skills would not affect the results by applying the ITPA subset “verbal expression” as a screening process. However, no scoring cut-off for this subset was suggested by the ITPA's authors. We opted for averaging the scores obtained by the sample and then subtracting the standard deviation. It is therefore possible that our screening is either too restrictive or may include subjects with slight language issues, but this cannot be ascertained without conducting a comparative analysis on an older population, which was beyond the scope of this study. That being said, we compared the data from children aged ≥8 years with that from children aged <8 and found no difference between the two groups in the perception of pain and somatisation. This indicates that the relationship between somatisation and emotional awareness is established precociously, likely in children even younger than those we examined.

Despite these limitations, this study lays the ground for further research into possible prevention strategies for somatisation, and in particular pain with somatoform characteristics. For instance, education about the recognition of emotions and awareness of the relationship between emotions and bodily sensations in primary school-age children could contribute to the prevention of somatisation and pain in later life.

## Figures and Tables

**Table 1:** Description of variables in the sample of primary school children.

		N	%	min	max	xM	sd
Age		445		6	10	7.82	0.65
Age ≥ 8 year	Yes	306	68.80				
	No	139	31.20				
Gender	Males	242	54.40				
	Females	203	45.60				
Primary school classes	II	154	34.60				
	III	270	60.70				
	IV	21	4.70				
No Italian	Yes	24	6.40				
	No	421	94.60				
KidsScreen 10	Total	445	92.97	13	50	32.90	6.34
Children's Somatization Inventory (CSI)	Global	445	91.94	0	40	14.890	8.63
Item 5	Back Pain (BP)	167	37.52				
Item 3	Chest Pain (CP)	118	26.51				
Item 24	Limb Pain (LP)	250	56.17				
Item 15	Stomach, Abdominal Pain (AP)	244	54.83				
Item 1	Headache (H)	259	58.20				
Item 6	Sore muscles (M)	134	30.11				
Total pain	At least 1 site	377	84.07				
	Severity of pain			0	3	1.54	0.93
	Number of sites			0	6	2.76	1.84
Levels of Emotional Awareness Scale for children (LEAS-C)							
	Self	445		16	43	32.07	4.56
	Other	445		16	42	30.89	4.76
	Total	445		19	52	34.14	4.39
Illinois Test of Psycholinguistic Abilities (ITPA)	Verbal expression subtest	445		23	52	30.02	5.26

**Table 2:** Correlation between emotional awareness, pain, QoL, and somatisation.

	Age	LEAS Self	LEAS Others	LEAS Total	Language	CSI	KidsScreen 10	Pain Intensity	WS	BP	CP	LP	AP	H	M
Age		0.14^*∗∗*^		0.13^*∗∗*^											
LEAS Self	0.14^*∗∗*^		0.64^*∗∗*^	0.87^*∗∗*^	0.13^*∗∗*^	-.11^*∗∗*^		-0.14^*∗∗*^		-0.12^*∗*^				-0.12^*∗*^	
LEAS Others		0.64^*∗*^*∗*		0.71^*∗∗*^											
LEAS Total	.13^*∗∗*^	0.87^*∗∗*^	0.71^*∗∗*^		0.18^*∗∗*^									0.13^*∗∗*^	
Language		0.14^*∗∗*^		0.18^*∗∗*^					0.14^*∗∗*^					0.13^*∗∗*^	0.11^*∗*^
CSI		-.11^*∗*^						0.72^*∗∗*^	0.72^*∗∗*^	0.42^*∗∗*^	0.46^*∗∗*^	0.60^*∗∗*^	0.46^*∗∗*^	0.65^*∗*^*∗*	0.43^*∗∗*^
Kidscreen 10		0.08^*∗*^							-0.11^*∗*^				-0.14^*∗∗*^	-0.11^*∗*^	
Pain Intensity		-0.14^*∗∗*^				0.72^*∗∗*^				0.42^*∗∗*^	0.31^*∗∗*^	0.60^*∗∗*^	0.49^*∗∗*^	0.55^*∗∗*^	0.36^*∗∗*^
Widespread pain (WS)					0.14^*∗∗*^	0.72^*∗∗*^	-0.11^*∗*^	0.60^*∗∗*^		0.53^*∗∗*^	0.60^*∗∗*^	0.52^*∗∗∗∗*^	0.50^*∗∗*^	0.62^*∗∗*^	0.56^*∗∗*^
Back pain (BP)		-0.12^*∗*^				0.42^*∗∗*^		0.42^*∗∗*^	0.53^*∗∗*^		0.28^*∗∗*^	0.23^*∗∗*^	0.16^*∗∗*^	0.31^*∗∗*^	0.20^*∗∗*^
Chest Pain (CP)						0.46^*∗∗*^		0.31^*∗∗*^	0.60^*∗∗*^	0.28^*∗∗*^		0.26^*∗∗*^	0.25^*∗∗*^	0.31^*∗∗*^	0.22^*∗∗*^
Limbs Pain (LP)						0.60^*∗∗*^		0.59^*∗∗*^	0.52^*∗∗*^	0.23^*∗∗*^	0.26^*∗∗*^		0.14^*∗∗*^	0.31^*∗∗*^	0.29^*∗∗*^
Abdominal Pain AP)						0.46^*∗∗*^	-0.14^*∗∗*^	0.50^*∗∗*^	0.50^*∗∗*^	0.16^*∗∗*^	0.25^*∗∗*^	0.14^*∗∗*^		0.32^*∗∗*^	0.22^*∗∗*^
Headache (H)		-0.11^*∗∗*^			0.13^*∗*^	0.65^*∗∗*^	-0.11^*∗*^	0.55^*∗∗*^	0.62^*∗∗*^	0.31^*∗∗*^	0.31^*∗∗*^	0.31^*∗∗*^	0.32^*∗∗*^		0.34^*∗∗*^
Sore Muscles (M)					0.11^*∗*^	0.44^*∗∗*^		0.36^*∗∗*^	0.56^*∗∗∗*^	0.20^*∗∗*^	0.22^*∗∗*^	0.29^*∗∗*^	0.22^*∗∗*^	0.34^*∗∗*^	

Coefficient of correlation r; ^*∗*^p< 0.05; ^*∗∗*^p< 0.01; ^*∗∗∗*^p< 0.001; ^*∗∗∗∗*^p< 0.0001.

**Table 3:** Beta standardized coefficient of the stepwise regression model.

Model			ITPA		CSI		BP		H		M		Severity Pain		widespread pain
	Predictors	R		R		R		R		R		R		R	
1	LEAS Self			.122	-.122^*∗*^	.132	-.132^*∗∗*^	.144	-.144^*∗∗*^			.161	-.161^*∗∗∗*^		

2	LEAS Total	.182	.182^*∗∗∗∗*^												

3	LEAS Self						-.490^*∗∗∗∗*^						-.379^*∗∗∗∗*^		
	LEAS Total					.247	.420^*∗∗∗∗*^					.204	.252^*∗*^		

4	LEAS Self														-.344^*∗∗∗*^

5	Leas Other														-.147^*∗*^
	LEAS Total														.388^*∗∗∗∗*^
	ITPA													.245	.117^*∗*^

6	LEAS Self								-.160^*∗∗*^		-.118^*∗*^				-.100^*∗*^
	ITPA							.204	.145^*∗∗*^	.159	.120^*∗*^			.169	.147^*∗∗*^

7	LEAS Self								-.355^*∗∗∗∗*^						-.350^*∗∗∗∗*^
	LEAS Total								.227^*∗*^						.290^*∗∗*^
	ITPA							.233	.130^*∗*^					.221	.127^*∗*^

8	ITPA									.107	.107^*∗*^			.136	.136^*∗∗*^

ITPA: Illinois Test of Psycholinguistic Abilities, verbal expression subtest; CSI: Children's Somatization Inventory.

BP: back pain, H: headache; M: sore muscle.

^*∗*^p<0.05; ^*∗∗*^p<0.05; ^*∗∗∗*^p<0.001; ^*∗∗∗∗*^p<0.0001

## Data Availability

The data used to support the findings of this study are included within the article and in the supplemental materials.

## Supplementary Materials

Supplementary Materials Table showing mean (sD) of all variables related to the original sample of 484 elementary schoolchildren used to support the results of this study. The variables studied have been divided by gender and age.
